# Comparación del resultado del movimiento dentario ortodóncico usando alineadores versus ortodoncia fija: una revisión

**DOI:** 10.21142/2523-2754-1102-2023-154

**Published:** 2023-06-29

**Authors:** Mónica Ivette Malpartida-Pacheco, Julissa Amparo Dulanto-Vargas

**Affiliations:** División de Ortodoncia, Universidad Científica del Sur. Lima, Perú. 100069885@cientifica.edu.pe Universidad Científica del Sur División de Ortodoncia Universidad Científica del Sur Lima Peru 100069885@cientifica.edu.pe; 2 Research Group in Dental Sciences, Carrera de Estomatología, Universidad Científica del Sur. Lima, Perú. jdulanto@cientifica.edu.pe Universidad Científica del Sur Carrera de Estomatología Universidad Científica del Sur Lima Peru jdulanto@cientifica.edu.pe

**Keywords:** movimientos ortodónticos, alineadores, *brackets* convencionales, ortodoncia fija, orthodontic movements, aligners, conventional brackets, fixed orthodontic

## Abstract

**Introducción:**

Los alineadores se han vuelto una alternativa de preferencia en cuanto al tratamiento de ortodoncia y han superado la elección de *brackets* convencionales, debido a la comodidad y estética que representan; sin embargo, sigue siendo un tema controversial el resultado final con este sistema. El objetivo de esta revisión sistematizada fue verificar la efectividad del tratamiento finalizado con alineadores versus con *brackets* convencionales.

**Materiales y métodos:**

Se realizó una búsqueda exhaustiva en las bases de datos de PubMed, ScienceDirect, Scopus y Embase hasta la fecha del 5 de enero de 2023. Se incluyeron estudios comparativos que evaluaron el resultado final y el tiempo de tratamiento de los alineadores en comparación con los *brackets* convencionales. Dos investigadores seleccionaron cuidadosamente los artículos evaluados y analizaron diferentes tópicos clave sobre el tema.

**Resultados:**

En este estudio se incluyeron 8 artículos; según los estudios, la gran mayoría no encontraron diferencias significativas en la finalización entre los grupos comparados. Adicionalmente, el tiempo de tratamiento en 3 artículos fue más rápido con alineadores, en 3 artículos fue más rápido con *brackets* convencionales y en un artículo no se encontró diferencia significativa, por lo que aún no está establecido si un sistema es más rápido que el otro.

**Conclusiones:**

Tanto alineadores como ortodoncia fija convencional tuvieron buenos resultados en la finalización del tratamiento de ortodoncia y presentaron un tiempo de tratamiento semejante; sin embargo, la mayoría de casos tratados pertenecieron a maloclusiones de mediana a baja complejidad, por lo que no se puede generalizar estos hallazgos a todas las maloclusiones.

## Abstract (en)

**Introduction:**

The aligners have become a preferred alternative in terms of orthodontic treatment, surpassing the choice of conventional brackets, due to the comfort and aesthetics that the aligners represent, however, the final result with this system continues to be a controversial issue. The objective of this review was to verify the effectiveness of the treatment completed with aligners versus conventional brackets.

**Materials and methods:**

An exhaustive search was carried out in the PubMed, ScienceDirect, Scopus and Embase databases up to the date of January 5, 2023, including comparative studies that evaluated the final result and treatment time of the aligners compared to conventional braces. Two researchers carefully selected the articles evaluated and analyzed different key topics on the subject.

**Results:**

In this study, 8 articles were included, according to the studies, the vast majority did not find significant differences in completion between the compared groups. In addition to this, the treatment time in 3 articles was faster with aligners, in 3 articles it was faster with conventional brackets, and in one article no significant difference was found, so it is not yet established whether a system is faster than the other.

**Conclusions:**

Both aligners and conventional fixed orthodontics had good results at the end of orthodontic treatment and presented a similar treatment time, however, the majority of treated cases belonged to malocclusions of medium to low complexity, so these cannot be generalized. findings to all malocclusions.

## INTRODUCCIÓN

Actualmente, existe una alta demanda de tratamientos ortodóncicos en los pacientes adultos; el uso de arcos, ligaduras, aparatos ortopédicos y otros elementos de la ortodoncia convencional dificultan la higiene dental, además que interfieren con la estética y causan incomodidad al paciente ^1^^-^^4^. Los alineadores transparentes se han convertido en una importante alternativa estética; sin embargo, se han observado limitaciones con este tipo de tratamiento en cuanto a su eficacia con ciertas maloclusiones y algunos movimientos, como torque, cambios sagitales, resalte, cierre de espacios por exodoncias, entre otros ^5^^,^^6^. 

El uso de alineadores es adecuado para grados de apiñamiento bajos a moderados (1-6 mm), para espacios medianos a moderados (1-6 mm) y para la recidiva después de la terapia de ortodoncia convencional ^7^^-^^9^. Las desventajas relacionadas con el uso de alineadores transparentes incluyen control limitado sobre el movimiento de la raíz, a diferencia de los *brackets* convencionales ^10^.

Un estudio en 2005 informó que los alineadores transparentes y el tratamiento con *brackets* convencionales lograron puntajes similares para la angulación de la raíz al final del tratamiento ^11^. En 2007, otro estudio encontró un empeoramiento estadísticamente significativo con respecto a la estabilidad de la alineación total después de alineadores transparentes, en comparación con el tratamiento con ortodoncia después de 3 años de retención ^12^. En general, se piensa que los alineadores pueden inclinar fácilmente las coronas, pero no las raíces debido a la falta de control del movimiento de los dientes ^13^. Esta conclusión es reforzada por Drake *et al*. ^14^, quienes afirman que el movimiento corporal no se puede lograr con los alineadores. Por el contrario, Simon *et al*. ^15^ reportaron una alta precisión (88%) del movimiento en cuerpo entero de los molares superiores cuando se indicó un movimiento de distalización de por lo menos 1,5 mm. 

Actualmente, hay una falta de literatura sobre los movimientos precisos con alineadores transparentes en comparación con los *brackets* convencionales, ^16^^-^^18^ específicamente en la finalización y el tiempo de tratamiento. Por ello, este estudio tuvo como propósito comparar el resultado del movimiento dentario ortodóncico en la finalización de un tratamiento de ortodoncia expresado en el torque final, la angulación de raíces y la posición vertical y el tiempo total de tratamiento usando alineadores versus ortodoncia fija.

## MATERIALES Y MÉTODOS

### Estrategia de búsqueda

La búsqueda de la bibliografía se llevó a cabo en las siguientes bases de datos: PubMed, Scopus, Embase y ScienceDirect, hasta el 5 de enero de 2023. La estrategia se basó siguiendo la estrategia PICOS: “P” (Población), pacientes adultos con maloclusiones o apiñamiento; “I” (Intervención), tratamiento ortodóncico con alineadores; “C” (Comparación), tratamiento ortodóncico con *brackets* convencionales; “O” (Resultados), sobre la finalización del tratamiento con ortodoncia en los movimientos dentarios como torque, angulación de raíces, y posición vertical y tiempo total de tratamiento; y “S” (diseños de estudio) observacionales y ensayos clínicos. 

La estrategia de búsqueda consideró descriptores MeSH y palabras claves en los títulos o resúmenes, utilizando los siguientes operadores booleanos: outcome, assessment, efficacy, effectiveness, accuracy evaluation, movements, stability, clear appliances, clear aligner, invisalign, brackets, conventional y fixed. Las referencias fueron estructuradas para impedir la réplica a través del gestor de referencias Refworks (Tabla 1).

**Tabla 1:** Estrategia de búsqueda de descriptores de las diferentes bases de datos

PubMed (05/01/2023)
n = 91
(comparison[Title/Abstract] OR outcome[Title/Abstract] OR assessment[Title/Abstract] OR efficacy[Title/Abstract] OR effectiveness[Title/Abstract] OR accuracy[Title/Abstract] OR evaluation[Title/Abstract] OR movements[Title/Abstract] OR stability[Title/Abstract]) AND ("clear appliances"[Title/Abstract] OR “clear aligner”[Title/Abstract] OR invisalign[Title/Abstract]) AND (brackets[Title/Abstract] OR conventional[Title/Abstract] OR fixed[Title/Abstract])
Scopus (05/01/2023)
n = 166
TITLE-ABS-KEY ((comparison OR outcome OR assessment OR efficacy OR effectiveness OR accuracy OR evaluation OR movements OR stability) AND (“clear appliances” OR “clear aligner” OR invisalign) AND (brackets OR conventional OR fixed)) AND (LIMIT-TO (DOCTYPE, "ar"))
Embase (05/01/2023)
n = 89
(comparison:ti,ab,kw OR outcome:ti,ab,kw OR assessment:ti,ab,kw OR efficacy:ti,ab,kw OR effectiveness:ti,ab,kw OR accuracy:ti,ab,kw OR evaluation:ti,ab,kw OR movements:ti,ab,kw OR stability:ti,ab,kw) AND ('clear appliances':ti,ab,kw OR 'clear aligner':ti,ab,kw OR invisalign:ti,ab,kw) AND (brackets:ti,ab,kw OR conventional:ti,ab,kw OR fixed:ti,ab,kw)
ScienceDirect (05/01/2023)
n = 18
Title, abstract, keywords: (comparison OR assessment OR efficacy OR effectiveness) AND (“clear appliances” OR "clear aligner" OR invisalign) AND (brackets OR fixed) | Refine by: Research articles

### Criterios de búsqueda

Los criterios de inclusión fueron investigaciones sobre tratamientos ortodóncicos en adultos de 18 a 35 años; investigaciones transversales o longitudinales, comparativos tipo ensayos clínicos, cohortes y casos y control; todas estas investigaciones contrastaron resultados de finalización entre alineadores y *brackets* convencionales metálicos o estéticos, vistas con radiografías, tomografías o modelos de estudio. Las investigaciones fueron publicadas en idioma inglés. Se excluyeron estudios en individuos con *brackets* autoligantes, enfermedades o síndromes, así como las revisiones narrativas, revisiones sistemáticas o metaanálisis, estudios descriptivos, cartas al editor, editoriales, artículos de opinión o reporte de caso(s), estudios duplicados o formatos con texto incompleto (“*no full text*”).

### Recolección de datos

**Figura 1:**
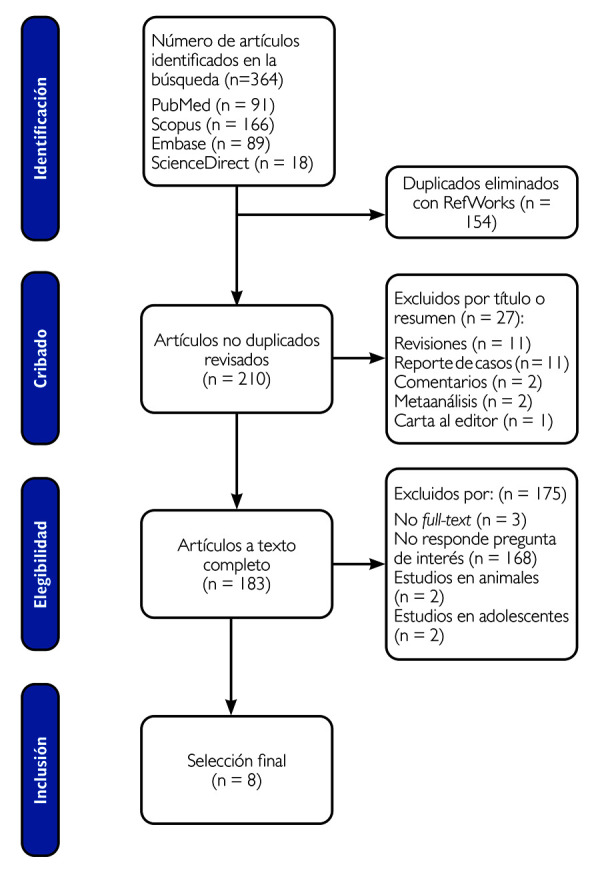
Diagrama de flujo PRISMA de la revisión de la literatura

La investigadora (MIMP) se calibró con un segundo investigador (JD) para revisar un total de 10 artículos con los criterios de selección, y se obtuvo un 100 % de concordancia. Las investigaciones recabadas se revisaron con base a títulos y resúmenes según los criterios de selección (MIMP). En el caso de que alguna investigación no presentara un resumen, se indagó el texto completo. Las disyuntivas o problemas de la recopilación fueron absueltas por un segundo investigador (JD). La información de los artículos y las razones para excluirlos fueron registradas en una base de datos. La manera de elegir los artículos siguió el método PRISMA (Preferred Reporting Items for Systematic Reviews and Meta-Analyses) (Figura 1).

### Resultados de interés

La investigadora (MIMP) sintetizó la información de las investigaciones elegidas y los datos extraídos de los artículos fueron los siguientes: autor, país, año de publicación, diseño de estudio, tamaño de muestra (sexo), rango de edad, características clínicas, intervención de interés (tipo de aparatología, tratamiento con o sin extracciones, y tiempo de tratamiento), instrumento de medición, dientes evaluados, resultados descriptivos e inferenciales.

## DESARROLLO

Identificación de los estudios: En la búsqueda se identificaron 364 artículos distribuidos en PubMed (91), Scopus (166), Embase (89) y ScienceDirect (18), y se encontraron 154 duplicados con Refworks. De los 210 artículos no duplicados, se excluyeron 27 por título o resumen (11 reporte de casos, 11 revisiones, 2 comentario, 2 metaanálisis y 1 carta al editor) y 183 en la revisión a texto completo, de los cuales 168 no respondieron la pregunta de interés, 3 aparecieron como “*no full text*”, 2 estudios se realizaron en animales y 2 estudios, en adolescentes. Finalmente, fueron seleccionados 8 artículos.

Datos demográficos y clínicos: Todos los estudios fueron publicados entre los años 2005 y 2022, y aplicados a 3 poblaciones: 5 en EE. UU. ^8^^,^^11^^,^^12^^,^^17^^,^^19^, 2 en Italia ^16^^,^^18^ y 1 en China ^20^. Los diseños de los estudios fueron 3 retrospectivos ^17^^,^^18^^,^^20^, 2 cohortes retrospectivos ^11^^,^^12^, 2 casos y control, 1 retrospectivo ^19^, 1 prospectivo ^16^ y 1 ensayo clínico aleatorizado ^8^. El rango de la muestra osciló de 22 a 96 pacientes en total y de 11 a 48 por tipo de aparatología ortodóncica. La distribución de pacientes por diseños de estudio fue retrospectiva, de 38 hasta 53; cohortes, de 22 y 96; casos y control, de 24 y 96; ensayo clínico aleatorizado, con 66, y prospectivo, con 44. Solo 2 estudios no refirieron la distribución por sexo ^11^^,^^18^ y 1 estudio brindó la distribución de sexo sin especificar el tipo de aparatología ^18^. El rango de muestra de las mujeres evaluadas fue de 8 a 32 y el de los varones fue de 1 a 18. La distribución de pacientes por tipo de aparatología resultó en alineadores de 10 a 32 mujeres y de 1 a 16 varones, y en *brackets* convencionales, de 8 a 30 mujeres y de 1 a 18 varones. Solo un estudio no brindó la edad según tipo de aparatología ^18^. La media de edad osciló entre 19 y 35 años en alineadores y de 18 a 32 en *brackets* convencionales. Los criterios de inclusión que se manejaron fueron pacientes sin extracciones previas ^8^^,^^11^^,^^12^^,^^16^^-^^20^, apiñamiento leve ^8^^,^^18^, moderado ^11^^,^^12^^,^^17^^,^^20^ y severo ^16^. Solo un estudio no precisó la maloclusión ^19^.

**Tabla 2:** Tabla de extracción de información

Id	Autor, país, año	Diseño de estudio	Tamaño de muestra (n de mujeres y n de hombres)	Rango de edad	Características clínicas	Intervención de interés		Instrumento de medición	Dientes evaluados	Resultados descriptivos	Resultados inferenciales
						Tipo de aparatología y tratamiento	Tiempo de intervención				
1	Garret *et al*., EE. UU., 2005	Cohortes retrospectivo	Total 96: BC (n =48), IN (n = 48) [NR sexo]	IN (33,6 ± 11,8); BC (23,7 ± 11,0)	Complejidad moderada según ID de ABO	IN / BC sin extracciones	IN 16 meses / BC 19 meses	OGS (Aprobado ≤30, desaprobado >30)	Todos en general según ABO	Puntaje total: IN (-45,35) BC (-32,21) Grupos con diferencia estadísticamente significativa (p <0,05) fue de IBL (IN-4.19 ±2.73; BC -2.81 ±2.63), COC (IN -10.46±7.06; BC -5.65 ±4.66), RO (IN -7.71 ± 4.76; BC -5.50 ± 4.71) y OJ (IN -6.21 ± 4.64; -3.56 ± 2.54). IN (10 aprobados y 38 reprobados) BC (23 aprobados y 25 reprobados)	Según OGS el resultado final entre IN y BC obtuvieron diferencias significativas (p<0,05) Obteniendo mejor puntaje los BC
2	B. Garnett *et al*., EEUU, 2018	Retrospectivo	Total 53: BC (n = 17: H = 9, M = 8), IN (n = 36: H = 9, M = 27)	IN (35.3 ± 7.3); BC (32.8 ± 11.9)	Hiperdivergentes con mordida abierta anterior	IN /BC [NR extracciones]	IN 18 ± 6 meses / BC 18 ± 7 meses	Análisis cefalométrico	Caninos e incisivos	OB (IN de -1.57 ± 1.24 a 0.71 ± 0.94) (BC de -1.3 ± 1.22 a 0.46 ± 0.93)	No hay diferencias significativas en el control de la dimensión vertical entre IN y BC en pacientes hiperdivergentes con mordidas abiertas anteriores.
3	F. Gaffuri *et al*., Italia, 2020	Casos y control prospectivo	Total 24: BC (n = 24: H = 11, M = 13), IN n: 12; BC n:12)	IN (Media 19); BC (Media 18)	Apiñamiento severo o maloclusión bimaxilar, dentición permanente sin previa ortodoncia	IN / BC (ambos con extracciones)	IN 29 meses / BC 24 meses	OGS (Aprobado ≤30, desaprobado >30)	Todos en general según ABO	Puntaje total medio: IN -17; BC -16 (ambos aprobados)	Según OGS, el resultado final entre IN y BC no obtuvieron diferencias significativas (p>0,05)
4	H. Chen *et al*., China, 2022	Retrospectivo	Total 38: BC (n = 20: H = 9, M = 11), IN (n = 18: H = 6, M = 12)	BC 23,60 ± 3.19; IN 22,67 ± 3,12 Años	Maloclusión moderada clase II div 2 con OB aumentado, apiñamiento de 0-8mm	IN / BC (no precisa extracciones)	IN 30.94 ± 4.32 meses / 28.30 ± 5.08 meses	Análisis tomográfico sagital	Incisivos superiores	IBL (IN 46,60 ± 1,70; BC 47,07 ± 3,24; BA 47,47 ± 2,15) p < 0,0001	Todos los sistemas tuvieron una IBL hacia adelantes y arriba, sin embargo, no obtuvieron diferencias significativas.
5	D. Kuncio *et al*., EEUU, 2007	Cohorte retrospectivo	Total 22: BC (n = 11: H = 1, M = 10), IN (n = 11: H = 1, M = 10)	IN (33,97 ± 8,98), BC (26,79 ± 12,12)	complejidad moderada según ID de ABO	IN / BC (ambos sin extracciones)	IN 20 meses / BC 28 meses	OGS (Aprobado ≤30, desaprobado >30)	Todos en general según ABO	Puntaje total: IN(-39.45 ±10.26) BC(-43.00±12.52) p 0.4760 IB (IN -3,45 ± 2,07; BC -2,81 ± 2,40); COC (IN -8.27 ± 4.24; BC -9.72 ± 5.02) RO (IN -6.73 ±4.64; BC -6.90 ± 4.83) OJ (IN -7 ± 3.79; BC -5.45 ± 4.29) AN (IN -2.09 ± 1.44; BC -2.09 ± 1.70) p valor 0.4760	El resultado final IN y BC si obtuvieron diferencias significativas obteniendo mejor puntaje los BC. Se evaluó la recidiva obteniendo peor puntaje los IN
6	E. Lin *et al*., EEUU, 2021	Ensayo controlado aleatorizado	Total 66: BC (n = 34), IN (n = 32) H=24, M = 42	IN (26,7 ± 9,8), BC (25,9 ± 16,6)	Maloclusión clase l molar y canino, apiñamiento inferior	IN / BC (ambos sin extracciones)	IN 19 meses / BC 15 meses	OGS (Aprobado ≤30, desaprobado >30)		Puntaje de Mediana IN 12, BC 17; CM(IN 2.0; BC1.0), IBL(IN 2.0; BC 2.0), COC (IN 1.0; BC 2.0), OJ (IN 1.0; BC 2.5), AN (IN 0.0, BC 1.0) RO (IN 2.0, BC 2.0)	Según OGS el resultado final entre IN tuvo mejores puntajes en COC, OJ y AN; y BC mejor puntaje en CM y RO, pero no obtuvieron diferencias significativas (p<0,05)
7	M. Sfondrini *et al*., Italia, 2018	Retrospectivo	Total 50: BC (n = 25), IN (n = 25) [NR sexo]	25,5 ± 6,5 años	Maloclusión clase l, o clase leve II y III con necesidad de torque incisal	IN / BC (no precisa extracciones)	No precisa	Análisis cefalométrico	Incisivos centrales	IBL (IN 5,13 ± 3,23; BA 5,64 ± 3,27; BC 6,11 ± 3,91) p > 0,05	Los BC resultaron con más IBL sin embargo no tuvieron diferencias significativas entre los 3 grupos.
8	Jiafeng Gu *et al*., EEUU, 2016	Casos y controles retrospectivo	Total 96: BC (n = 48: H=18, M=30), IN (n = 48: H = 16, M = 32)	IN 26.0 ± 9.7, BC 22.1 ± 7.9	Maloclusión (no refiere tipo)	IN / BC (no precisa extracciones)	IN 13.35 meses / BC 19.0 meses	índice PAR	Todos en general	Puntuación de gran mejora IN 22.90%, BC 45.8% (RM IN 0.02 ± 0.14, BC -0.02 ±0.14; OJ IN 5 ±4.85, BC 4.88 ±6.27; OB IN 1.35 ±1.76, BC 2.38 ±2.53)	La puntuación PAR media ponderada posterior al tratamiento de IN fue superior a la de BC, esto no fue estadísticamente significante.

IN (Invisalign), BC (*brackets* convencionales); ID (Índice de discrepancia) 7-15 leve, 16-24 moderada, >25 grave, ABO (American Boards of Orthodontics) OGS (Sistema de calificación objetiva)< o =30 puntos es aprobatorio; IBL (Inclinación bucolingual o torque), AN (Angulación radicular), RO (relaciones oclusales), COC (Contactos oclusales), OJ (Resalte), CM (Cresta marginal o posición vertical), OB (sobremordida), índice PAR mejora aceptada 22%; NR (No refiere) índice PAR (PAR ponderado del Reino Unido, que incluye el índice mandibular anterior) evalúa 8 componentes: alineación del segmento anterior maxilar, segmento anterior mandibular, alineación, discrepancia anteroposterior, discrepancia transversal, discrepancia vertical, resalte, sobremordida y línea media

Todos los estudios utilizaron la marca Invisalign® ^8^^,^^11^^,^^12^^,^^16^^-^^20^ en alineadores transparentes. Asimismo, todos los estudios evaluaron *brackets* metálicos; cuatro estudios no precisaron la marca y los demás incluyeron las siguientes: tip-edge fixed appliances (TP Orthodontics, LaPorte, Ind)^11^, Trademark of 3M, Monrovia, CA ^16^; Radiance MBT (American Orthodontics, Sheboygan, WI) en arco superior y prescripción de Alexander en arco inferior ^8^, y 2 de Victory Series (3M Unitek, Calif) ^18^^,^^20^. Tres estudios aplicaron tratamientos sin extracciones ^8^^,^^11^^,^^12^, uno con extracciones ^16^ y cuatro no precisaron si se realizaron o no extracciones ^17^^-^^20^. El tiempo de intervención fue referido por la mayoría de los estudios, con excepción de uno ^18^. En el caso de los alineadores, se intervino de 13 a 31 meses, y en *brackets* convencionales metálicos, de 15 a 28 meses. 

Los instrumentos de medición para evaluar los resultados de finalización del tratamiento de ortodoncia fueron los siguientes: 4 con OGS (sistema de calificación objetiva) ^8^^,^^11^^,^^12^^,^^16^, 3 análisis cefalométricos ^17^^,^^18^^,^^20^ y 1 análisis de índice PAR ^19^. Las medidas de la OGS son una herramienta para calificar numéricamente los modelos finales y las radiografías panorámicas enviadas para el examen de Fase III. Debido a que la certificación ABO es el logro más alto en las credenciales de ortodoncia, el uso de este método es el mejor para evaluar el resultado del tratamiento final. Sus medidas se dividen en alineación, crestas marginales, inclinación bucolingual, contactos oclusales, relaciones oclusales, resalte, contactos interproximales y angulación radicular. El número de puntos perdidos se sumó para obtener la puntuación OGS. Un caso que pierde 30 puntos o menos, generalmente, recibe una calificación aprobatoria para el examen ABO. En el análisis radiológico, se consideró el ángulo del eje del incisivo central superior con el plano palatino ^17^^,^^18^^,^^20^. 

El índice PAR se utilizó en un estudio (^19^) para evaluar 8 componentes: alineación del segmento anterior maxilar, alineación del segmento anterior mandibular, discrepancia anteroposterior, discrepancia transversal, discrepancia vertical, resalte, sobremordida y línea media.

## DISCUSIÓN

En la actualidad, la estética es una de las principales preocupaciones de los pacientes que desean iniciar un tratamiento de ortodoncia^5^; por ellos existen en el mercado distintas opciones de aparatos como *brackets* estéticos de zafiro o cerámica, *brackets* linguales y especialmente los alineadores ^21^. En este sentido, una ventaja de los alineadores, respecto de los otros métodos, es la capacidad de retirarse los alineadores para comer, cepillarse los dientes y usar el hilo dental; por lo tanto, tendrían mejor higiene, mejor salud periodontal y recuentos de bacterias orales más bajos, así como la comodidad, la facilidad de uso y la ausencia de lesiones dentro de la mucosa oral ^6^^,^^12^^,^^18^^,^^22^^-^^29^. Además, se ha reportado que los pacientes con alineadores presentan una reabsorción radicular menos grave que con la ortodoncia fija ^19^. 

Se ha cuestionado la eficacia y eficiencia de estos aparatos en comparación con la ortodoncia fija; sin embargo, según algunos autores ^1^^,^^11^^-^^13^, la finalización no fue tan buena en algunos movimientos específicos. Otros autores ^16^^,^^17^ informaron excelentes resultados en casos complejos como mordidas profundas, mordidas abiertas, extracción de premolares y corrección de clase II en crecimiento. La presente revisión busca evidencia científica para evaluar la efectividad de los alineadores invisibles en comparación con la ortodoncia fija convencional, ya que el conocimiento de sus capacidades y limitaciones es de suma importancia para realizar un diagnóstico adecuado y ofrecer un plan de tratamiento apropiado ^30^^-^^32^.

Todos los estudios seleccionados en esta revisión dieron a conocer el tiempo de tratamiento, menos uno ^18^. El rango de alineadores fue de 13 a 30 meses, y con la ortodoncia convencional fija, es decir, con los *brackets* convencionales, fue de 15 a 28 meses. Se observó en tres estudios ^1^^,^^5^^,^^19^ que finalizaron el tratamiento más rápido con alineadores transparentes; tres estudios ^3^^,^^6^^,^^20^ finalizaron el tratamiento más rápido con *brackets* convencionales; y un estudio ^2^ obtuvo casi el mismo tiempo de finalización en ambos métodos (alineadores: 18 ± 6 meses, *brackets*: 18 ± 7 meses).

Por otro lado, cuatro estudios ^8^^,^^11^^,^^12^^,^^16^ compararon los resultados del tratamiento finalizado de pacientes con Invisalign versus aparatos fijos usando el sistema de calificación objetiva (OGS), y se observó en alineación que solo un artículo obtuvo mejores resultados con *brackets* convencionales ^12^, y los demás artículos ^8^^,^^11^^,^^16^ encontraron valores similares. Respecto de la alineación de las crestas marginales, un artículo ^8^ obtuvo mejor respuesta con los *brackets* convencionales; por el contrario, otro artículo ^12^ obtuvo mejor respuesta con alineadores transparentes, y los otros dos artículos ^11^^,^^16^ encontraron puntajes similares. Además, con respecto a la inclinación bucolingual, los cuatro artículos obtuvieron mejores resultados con *brackets* convencionales. En resalte (*overjet*), solo un artículo ^8^ obtuvo mejor puntaje en los alineadores, a diferencia de los otros autores ^11^^,^^12^^,^^16^ en cuyos casos los *brackets* convencionales tuvieron mejor puntaje. Finalmente, la angulación radicular en un artículo ^8^ obtuvo mejor puntaje con los alineadores transparentes, en contraste con los otros artículos ^11^^,^^12^^,^^16^, que obtuvieron puntajes similares. 

Según otro estudio ^19^, que utilizó el índice de calificación de evaluación por pares (PAR), se obtuvo como resultado que el *overjet* fue mejor controlado con los *brackets* convencionales y, en general, la mejora fue más efectiva con ellos; no obstante, no se obtuvo diferencias significativas.

En cuanto a los pacientes con mordida abierta con ángulo alto, según un estudio de 2017 ^17^, se encontró que mejorar la sobremordida fue más fácil con alineadores que con *brackets* convencionales, y lo mismo ocurrió con la inclinación bucolingual (torque); sin embargo, no se obtuvo una diferencia significativa, por lo cual se puede lograr un buen control vertical usando cualquiera de los dos aparatos, cuando se planifica y ejecuta cuidadosamente la biomecánica. Por otro lado, dos estudios, de 2018 ^18^ y 2022 ^20^, encontraron que la inclinación bucolingual (torque) fue más controlada por los *brackets* convencionales, pero no obtuvieron diferencias significativas en comparación con los alineadores.

Todos los estudios ^8^^,^^12^^,^^16^^-^^20^ presentaron que no hay diferencias significativas en la eficacia del resultado de finalización entre *brackets* convencionales y alineadores transparentes, a excepción de un estudio ^11^ de 2005, el cual concluye que los *brackets* convencionales son más efectivos. Se puede presumir que, con las nuevas actualizaciones de Invisalign respecto de su protocolo G6 y con otros aditamentos, este consiga un mejor resultado en comparación con los estudios previos a 2010.

Existen algunas limitaciones, como que, a pesar que la ortodoncia fija y los alineadores pueden mover dientes, no hay literatura sobre cuánta fuerza se crea con los alineadores. Es importante recalcar que se pueden usar técnicas híbridas entre alineadores y dispositivos fijos para mejorar el resultado final ^11^. Por otro lado, debido a que los alineadores son removibles, el ortodoncista debe confiar en la motivación y confiabilidad del paciente para lograr el resultado deseado. La removibilidad de Invisalign es una ventaja para el paciente, pero no siempre para el ortodoncista ^11^. Se recomienda ampliar investigaciones con otras marcas de alineadores transparentes. Finalmente, debe quedar claro que la mayoría de las maloclusiones comparadas en las investigaciones eran de clase I y II, de mediana a baja complejidad, por lo que no se puede generalizar estos resultados a todas las maloclusiones y más investigaciones bien estructuradas metodológicamente deben realizarse. 

## CONCLUSIONES

El tratamiento ortodóncico con alineadores y *brackets* convencionales parece tener similar resultado en la finalización (torque, angulación de raíces y posición vertical) y tiempo total de tratamiento. Por tanto, es indispensable realizar un buen diagnóstico y plan de tratamiento, aunque cabe señalar que no se puede generalizar el éxito en todas las maloclusiones, especialmente en los casos de alta complejidad, ya que no han sido reportados en las investigaciones. 

## Fuente de financiamiento:

Esta investigación no posee fuentes de financiamiento.
